# A Rationally Designed Bovine IgA Fc Scaffold Enhances *in planta* Accumulation of a V_H_H-Fc Fusion Without Compromising Binding to Enterohemorrhagic *E. coli*

**DOI:** 10.3389/fpls.2021.651262

**Published:** 2021-04-14

**Authors:** Adam Chin-Fatt, Reza Saberianfar, Rima Menassa

**Affiliations:** ^1^Agriculture and Agri-Food Canada, London Research and Development Centre, London, ON, Canada; ^2^Department of Biology, University of Western Ontario, London, ON, Canada

**Keywords:** enterohemorrhagic *E. coli*-EHEC, IgA, V_H_H antibody fragment, single domain antibody (sdAb), Fc fusion, plant-made antibodies, transient expression, rational design antibody engineering

## Abstract

We previously isolated a single domain antibody (V_H_H) that binds Enterohemorrhagic *Escherichia coli* (EHEC) with the end-goal being the enteromucosal passive immunization of cattle herds. To improve the yield of a chimeric fusion of the V_H_H with an IgA Fc, we employed two rational design strategies, supercharging and introducing *de novo* disulfide bonds, on the bovine IgA Fc component of the chimera. After mutagenizing the Fc, we screened for accumulation levels after transient transformation in *Nicotiana benthamiana* leaves. We identified and characterized five supercharging and one disulfide mutant, termed ‘(5 + 1)Fc’, that improve accumulation in comparison to the native Fc. Combining all these mutations is associated with a 32-fold increase of accumulation for the Fc alone, from 23.9 mg/kg fresh weight (FW) to 599.5 mg/kg FW, as well as a twenty-fold increase when fused to a V_H_H that binds EHEC, from 12.5 mg/kg FW tissue to 236.2 mg/kg FW. Co-expression of native or mutated V_H_H-Fc with bovine joining chain (JC) and bovine secretory component (SC) followed by co-immunoprecipitation suggests that the stabilizing mutations do not interfere with the capacity of V_H_H-Fc to assemble with JC and FC into a secretory IgA. Both the native and the mutated V_H_H-Fc similarly neutralized the ability of four of the seven most prevalent EHEC strains (O157:H7, O26:H11, O111:Hnm, O145:Hnm, O45:H2, O121:H19 and O103:H2), to adhere to HEp-2 cells as visualized by immunofluorescence microscopy and quantified by fluorometry. These results collectively suggest that supercharging and disulfide bond tethering on a Fc chain can effectively improve accumulation of a V_H_H-Fc fusion without impacting V_H_H functionality.

## Introduction

Fragment crystallizable (Fc) fusion proteins represent 15% of all recently approved biologics by the United States Food and Drug Administration (FDA) (2011-2016), with significant market increases forecasted for 2016 to 2025 (Lagasse et al., 2017). Fc-fusion proteins, also known as Fc chimeric proteins or Fc-tagged proteins, comprise the Fc domain of an immunoglobulin (Ig) that has been genetically linked to a protein of interest. The appeal of the Fc as a fusion partner is afforded by its structural modularity allowing independent folding of its fusion partner while still providing structural stability (Czajkowsky et al., 2012). Additionally, the Fc domain homodimerizes with itself via an inter-chain disulfide bond allowing an increase in avidity for a fused partner (Bastian et al., 1992) and enabling effector functions (Park et al., 2016).

Although the market consists predominantly of IgG subtype Fc fusions, there has been much interest in developing an alternative IgA-based Fc scaffold for the control of mucosal pathogens in applications such as oral passive immunization (Bakema et al., 2011; Bakema and Van Egmond, 2011; Lohse et al., 2011; De Greve et al., 2020). Unlike the IgG Fc, the IgA Fc assembles with two other subunits, a 15 kDa joining chain (JC) and a 70 kDa secretory component (SC), to form a secretory complex (sIgA) (Supplementary Figure 1). Despite the therapeutic value of IgA, transition to clinical testing has been hampered by technical difficulties in producing and purifying the full-size IgA in conventional microbial and mammalian platforms because of native IgA’s large size (380 kDa; compared to 150 kDa for IgG) (Reinhart and Kunert, 2015). There are several reports of the production of IgA in plants and in yeast intended for oral passive enteromucosal immunization of animals (Virdi et al., 2013, 2019; Vanmarsenille et al., 2018; Saberianfar et al., 2019). While accumulation of IgA in some of those reports has met the 1% TSP/100 mg/kg FW/100 mg/L culture medium rule of thumb benchmark for commercial viability (Rybicki, 2009; Schillberg et al., 2019), an improvement in accumulation levels by way of stabilization would reduce the cost of production. According to a techno-economic analysis for monoclonal antibodies (mAbs) in a plant platform, an increase in accumulation level from 0.25 g/kg FW to 1 g/kg FW cuts production costs by almost 60% from ∼$290/g to ∼$120/g (Nandi et al., 2016). Therefore, there is value in exploring design strategies that improve IgA accumulation in a plant platform.

Structurally, IgA is a modular glycoprotein with an antigen binding fragment (Fab) linked to an Fc scaffold which consists of two dimerized constant domains, CH2 and CH3, that form interchain disulfides with the SC and JC respectively to enable assembly of the secretory complex (Supplementary Figure 1). To develop a stabilized IgA Fc chain that could improve yield of its fusion partner without impairing function, we investigated two rational design strategies, supercharging and introducing *de novo* disulfide bonds, on a bovine IgA Fc chain. The underlying idea behind both of these two designs is based on a number of previous proteomic observations that suggest that heterologous protein stability and solubility strongly correlate with protein abundance (Walther et al., 2015; Leuenberger et al., 2017). Conceptually, supercharging involves the addition of charged amino acids to the protein surface. Because these additional charges exhibit small charge-charge repulsive forces, protein accumulation can be enhanced by reducing the non-specific protein aggregation that typically occurs in the macromolecular crowded environment when producing recombinant proteins (Lawrence et al., 2007). Since such aggregates can be either refolded by chaperones, terminally sequestered or targeted for degradation via the proteasome (Mogk et al., 2018; McLoughlin et al., 2019), reducing the occurrence of non-specific aggregation may increase yield. The introduction of novel disulfides at strategic locations can enhance accumulation by preventing exposure of the reactive hydrophobic interior normally susceptible to proteolytic enzymes or non-specific aggregation to adjacent proteins (Zabetakis et al., 2014). The use of protease inhibitors to prevent degradation from proteolytic enzymes has been shown to improve heterologous mAb yield in plants (Jutras et al., 2016).

Enterohemorrhagic *Escherichia coli* (EHEC) has consistently been one of the foremost foodborne pathogen threats worldwide, conservatively estimated in causing 2.8 million acute illnesses annually (Majowicz et al., 2014). It primarily colonizes the bovine digestive tract as a reservoir from which it can be transmitted via fecal shedding or during slaughter to ultimately compromise food and water safety (Montenegro et al., 1990; Beutin et al., 1993). Collectively, seven EHEC strains (O157:H7, O26:H11, O111:Hnm, O145:Hnm, O45:H2, O121:H19 and O103:H2), account for over 95% of all EHEC-related illnesses and have been the main focus of North American food surveillance efforts for the past decade (Mathusa et al., 2010; Public Health Agency of Canada, 2015). Towards addressing this problem, we previously isolated camelid-derived single-domain antibodies (V_H_Hs) specific for intimin, an EHEC adhesin required for colonization, fused them to a bovine IgA Fc and demonstrated that these chimeric V_H_H-Fc fusions could bind and neutralize the adherence of three of the most prevalent EHEC serotypes: O157, O26, and O111 (Saberianfar et al., 2019). We subsequently showed that these chimeric fusions neutralize a 4th EHEC serotype, O145 (Chin-Fatt, unpublished). Simplified chimeric V_H_H-IgA Fc, which involve replacing the Fab region with a V_H_H, are much easier to produce and are encoded by a single gene, rather than four required for full sized sIgA (De Greve et al., 2020).

In this study, we identified a stabilized rationally designed bovine IgA Fc chain with five mutations predicted to supercharge the protein, and one mutation predicted to introduce a novel disulfide respectively. After transient transformation into *Nicotiana benthamiana* leaves, these mutations, when combined, improved *in planta* accumulation of both the Fc chain alone and of the Fc fused to an anti-EHEC V_H_H partner. Co-expression of the engineered V_H_H-Fc fusion with a bovine joining chain and secretory component and subsequent co-immunoprecipitation suggest that the mutations do not impair correct folding and assembly of components to the secretory IgA form. We also demonstrate that the engineered Fc fusion does not compromise the V_H_H’s function of binding and neutralizing EHEC strains O26:H11, O111:Hnm, O145:Hnm and O157:H7. This study is notable because it provides proof of concept that an Fc chain may be rationally designed for improved IgA yield in a plant platform without compromising functionality or its ability to assemble into its secretory form.

## Results

### Selection of Amino Acid Residues for Supercharging and Tethering of Unstructured Domains

We chose to work with the bovine Fc for neutralizing the pathogen by immune exclusion for several reasons. In the context of passive immunization there may be some activation of the host immune system that requires the bovine Fc. There is some evidence, though not conclusive, that secretory IgAs bound to pathogens in the gut associated lymphoid tissue (GALT) can pass through the epithelium and activate a subset of dendritic cells via the FcαR to upregulate CD86 costimulatory molecules, class II major histocompatibility complex (MHC) expression, and increased allostimulatory activity (Geissmann et al., 2001). We have intentionally not mutated the residues involved in this interaction. As well, using a bovine sequence reduces the chance of an allergic reaction by the host and may be more acceptable to regulatory approval than a non-bovine Fc.

**FIGURE 1:**
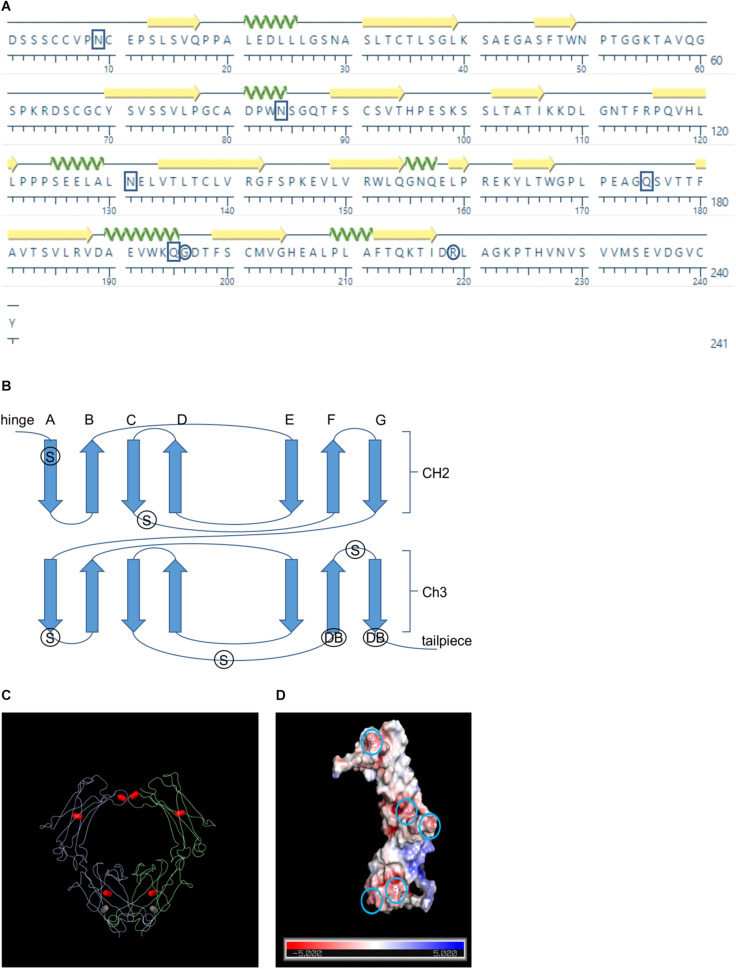
Schematics showing the positions of the rational design candidates. **(A)** Amino acid sequence of the bovine IgA Fc sequence. Boxes indicate positions of the candidates for supercharging and circles indicate the positions of the candidates for *de novo* disulfide bonds **(B)** Schematic showing the Greek key connectivity of the Fc’s β barrel structure. Arrows indicate β strands, S indicates the positions of supercharging candidates and DB indicates the positions of *de novo* disulfide bond candidates **(C)** Wire diagram of a dimerized Fc with native intra- and inter- chain disulfides colored in red and the *de novo* disulfide colored in gray **(D)** Surface representation of the bovine Fc chain with predicted charge colored on a scale of red indicating more electronegative to blue indicating more electropositive. Circles indicate the positions of the supercharging candidates

Although the crystal structure of human IgA Fc has been determined and is publicly available (pdb: 1IGA) (Boehm et al., 1999), bovine IgA Fc, which is 70% similar in sequence, has not yet been documented (Figure 1A). Because the structure of the Fc is generally well conserved across species, we hypothesized that we could use the human Fc for homology-based structural prediction of the bovine Fc rather than modeling it *ab initio* (Figure 1B). Therefore, we used the I-TASSER online program (Zhang, 2008) to predict the structure of bovine IgA Fc using the human IgA Fc as a threading template (Wu and Zhang, 2008; Zhang, 2008). Because the resulting predicted structure had a high confidence score of 1.35 (given a range of -5 to 2), it may be sufficient for rational design of mutations. The predicted secondary structure of the bovine IgA Fc consists of a characteristic β sandwich of seven anti-parallel β strands for both its CH2 and CH3 domains. Both domains exhibit Greek key connectivity, a topological signature of β sandwiches, (ABED CFG) forming two distinct β sheets that fold over each other (Figure 1C). For both domains, an intra-chain disulfide in the center of the β sheet stabilizes the tertiary structure. There are also inter-chain disulfides adjacent to the hinge area that enable dimerization of the Fc.

To develop a more stable Fc chain that could act as a stabilization partner when fused to a V_H_H, we tested two different rational design strategies: (1) changing key surface residues to give a more electronegative net charge, and (2) introducing *de novo* intra-chain disulfide bonds to stabilize the internal structure (Figures 1A–C). A total of 12 mutants were assessed of which five supercharging (N9D, N84D, N131D, Q175E, Q195E) and one disulfide (G196C/R219C) mutants were advanced for further characterization (Supplementary Table 1).

Supercharging residue candidates were determined by modeling a predicted bovine IgA Fc structure using the I-TASSER and PyMol programs (Schrodinger, 2010; Yang et al., 2015) and then selecting the most surface-exposed asparagine and glutamine residues for mutating to their conservative but negatively charged counterparts: aspartic acid and glutamic acid respectively. Aspartic acid and glutamic acid are structurally similar to asparagine and glutamine, differing only in their exposed side chains, and these substitutions are usually well tolerated in maintaining tertiary structure. The asparagines and glutamines were also selected based on non-involvement in native glycosylation (residues 29, 228) or Fc α receptor (FcαR) binding (residues 26, 117, 151, 153, 156, 158, 202, 205, 214). The asparagines that were mutated are unlikely to be involved in N-glycosylation considering that the adjacent residues do not match the standard glycosylation motif (N-X-S/T where X is a non-proline residue) and crystallization studies of human IgA have not suggested otherwise.

For the selection of *de novo* intra-chain disulfide bonds, the predicted Fc structure was modeled and the disulfide candidates chosen either by manual inspection of the molecule in PyMol or by ranked selection based on stability recommendations using the Disulfide by Design 2.0 software (Supplementary Table 2) (Craig and Dombkowski, 2013). The G196C/R219C disulfide candidate was manually chosen based on neighboring proximity with the intent of tethering the C-terminal end of strand G to the N terminal end of strand F (Figure 1B). The tailpiece leading out from strand G is unstructured and contains a free S-H that natively forms a disulfide bond with the J chain in dimeric IgA. With the G196C/R219C mutation, we hypothesized that tethering the tailpiece to strand F could potentially stabilize the molecule by hiding any vulnerable hydrophobic regions that may become exposed as the strand becomes unstructured, and in so doing, prevent access by proteolytic enzymes. In particular, free floating unstructured tails are known to be preferentially loaded into the 26S proteasome apparatus for degradation (Inobe and Matouschek, 2014). Thus, tethering the tailpiece by use of G196C/R219C could potentially prevent proteasome access.

### Supercharging and Disulfide Introduction Improve Fc Accumulation in Transiently Transformed Leaf Tissue

**FIGURE 2:**
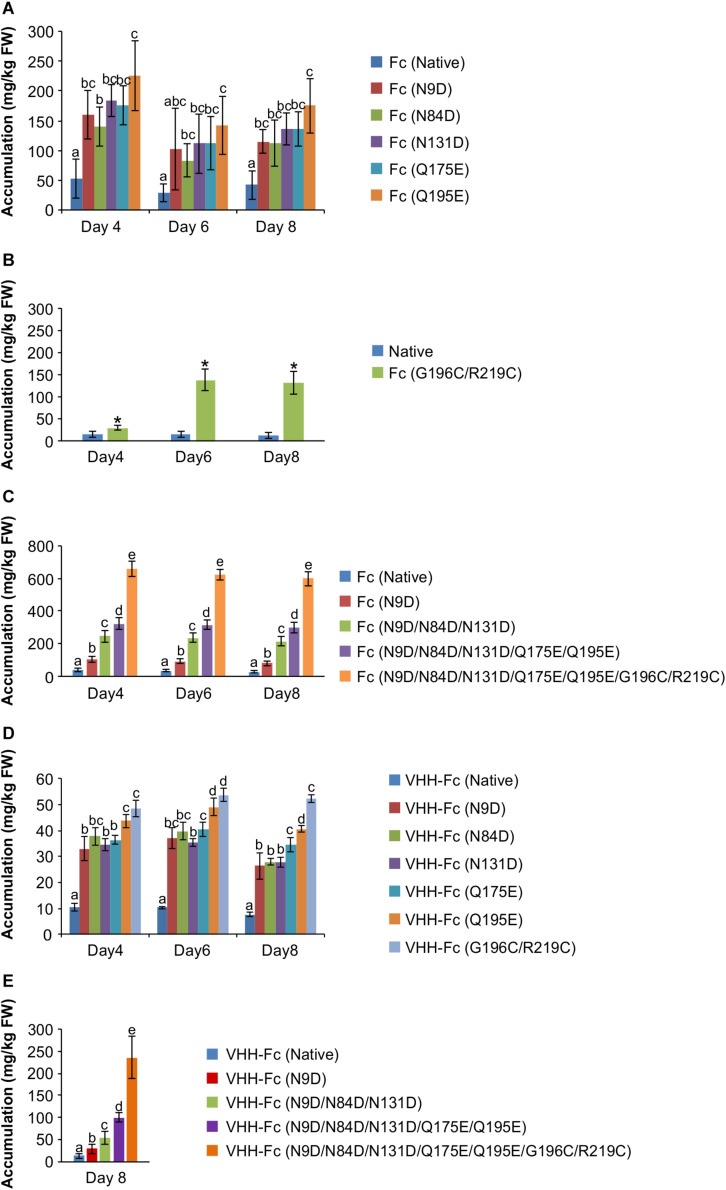
Time courses showing accumulation after transient transformation of *N. benthamiana* by agroinfiltration. Shown are **(A)** Supercharging mutations on the Fc chain. **(B)** Novel disulfides on the Fc chain **(C)** Combining of mutations on the Fc **(D)** Fc with individual mutations fused to a V_H_H **(E)** Fc with combined mutations fused to a V_H_H on 8 dpi. * represents statistically significant difference from native as determined by a *T*-test. Letters denote significantly different treatments as determined by one way ANOVA and *post hoc* Tukey HSD test. *P* < 0.05, *n* = 3-5 biological replicates. Error bars shown are standard error of the mean.

The rationally designed candidates were generated by site-directed mutagenesis of the native bovine IgA Fc and confirmed by sequencing. All candidates were then cloned into a pCaMGate plant expression vector for ER targeting and retrieval (Pereira et al., 2014). We chose to target the constructs to the ER because of the requirement for disulfide formation for correct folding and assembly and also because V_H_H-Fc accumulation has been previously shown to be robust (220 mg/kg FW) when targeted to the ER using the pEAQ plant expression vector (Sainsbury et al., 2009; Saberianfar et al., 2019). Screening of ER-targeted wild type and mutant Fc (Supplementary Table 1) was done by agroinfiltration of *N. benthamiana* leaves and semi-quantitative western blotting at four, six and eight days post-infiltration (dpi). Compared to accumulation of native Fc (42.30 ± 10.83 mg/kg), by day eight, each of the supercharged Fc mutants showed between a two- to four-fold improvement (between N84D at 112.39 ± 17.11 mg/kg to Q195E at 175.17 ± 20.22 mg/kg) that persisted across the time course post infiltration (Figure 2A, Supplementary Table 3). Of the six disulfide pairs tested (Supplementary Table 1), two (K40C/pre-existing cysteine at position 10, and G196C/R219C) improved yield by two- and 10-fold (35.91 mg/kg for K40C and 132.02 mg/kg for G196C/R219C) (Figure 2B). However, upon further examination of the predicted model of the bovine IgA Fc, we decided to abandon K40C because of the potential for distorting correct inter-chain disulfide formation at the hinge. To test if these mutations could be combined to further improve accumulation, we combined the mutations step-wise and measured accumulation in transformed leaf extract by western blot. Combining the mutations gave an incremental increase in accumulation after transient expression. The Fc construct containing all five supercharging residues showed a seven-fold improvement (296.18 ± 18.47 mg/kg) in accumulation compared to native (Figure 2C). Adding the *de novo* disulfide to these five supercharging residues further improved accumulation to an approximately 32-fold improvement (599.47 ± 25.40 mg/kg) (Figure 2C). To test if these Fc mutants could also enhance accumulation as an Fc fusion protein, we fused each to a V_H_H that was identified from panning a phage display library with intimin of O157:H7 (denoted as V_H_H9 in Saberianfar et al., 2019). Similar to the comparison using Fc alone, each of the mutant V_H_H-Fc fusions showed between a three to six-fold improvement (between 26.42 ± 4.96 mg/kg for N9D to 52.38 ± 1.39 mg/kg for G196C/R219C) in accumulation when compared to native (7.57 ± 0.89 mg/kg) by day eight (Figure 2D). When fused to the V_H_H, combining the mutations on the Fc incrementally improved accumulation; mutants with all five supercharging mutations plus the disulfide showed a twenty-fold improvement (236.19 ± 41.32 mg/kg) compared to native (12.45 ± 5.86 mg/kg) on day eight (Figure 2E). For consistency, day 8 post-infiltration was selected for extraction towards binding and neutralization assays. Because the engineered construct containing all supercharging and disulfide mutations (5 + 1 Fc) showed the most promise, we focused on it for further characterization.

### Engineered V_H_H-(5 + 1)Fc Assembles With Other sIgA Subunits *in planta*

**FIGURE 3:**
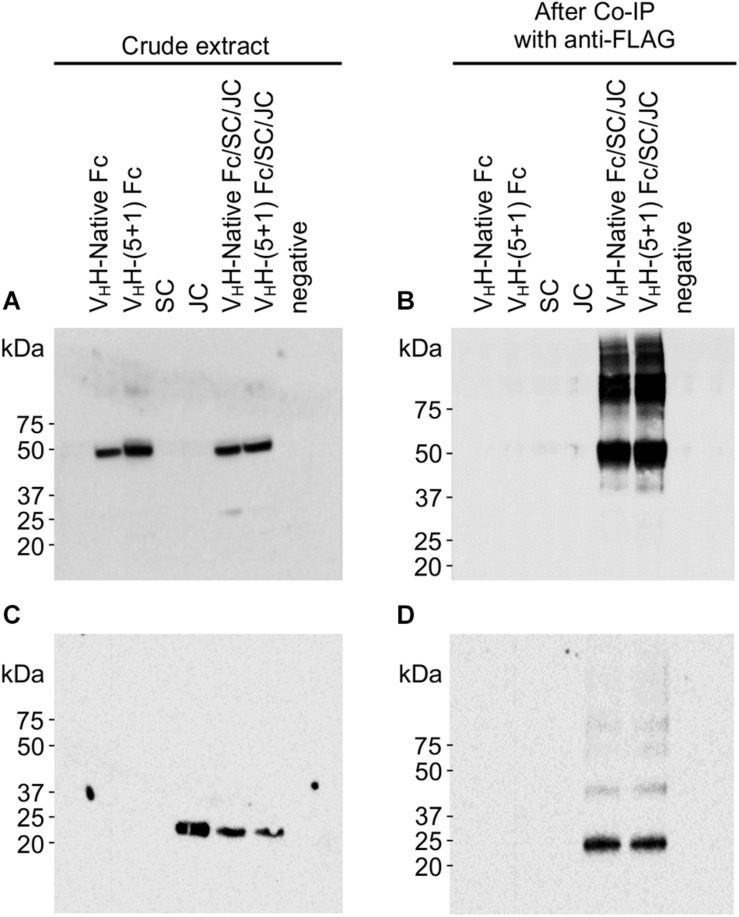
V_H_H-(5 + 1) Fc retains the ability to assemble with other secretory IgA subunits SC and JC. Shown are Western blots probed with either anti-cmyc **(A,B)** or anti-HA **(C,D),** which correspond to differently tagged subunits V_H_H-Fc and JC respectively. Leaf issue was transformed with constructs of each subunit individually and also with combinations of V_H_H-Fc/SC/JC and V_H_H-(5 + 1)Fc/SC/JC for intended co-expression and assembly. Detection was done for both diluted crude leaf extract **(A,C)** and for the eluent after the extract had been co-immunoprecipitated using an anti-FLAG column **(B,D)**.

Although the V_H_H-Fc fusion lacks the light chains and CH1 domains found in native mammalian sIgA, assembly to the JC and SC is directed specifically via disulfide bond formation with the Fc (Supplementary Figure 1). JC forms a disulfide bond with the tailpiece in the CH3 domain from opposite Fc chains orienting them end-to-end, and SC forms a disulfide bond with opposite CH2 domains from each Fc. To determine if engineering of the V_H_H-Fc affected its ability to assemble with the SC and JC subunits, we co-expressed all three subunits for immunoprecipitation experiments. The JC and SC sequences had previously been cloned into pEAQ-DEST-1 expression vectors (Sainsbury et al., 2009) with ER targeting and retrieval sequences added (Saberianfar et al., 2019). Each subunit is also linked to a different tag (V_H_H-Fc-c-myc; SC-Flag; JC-HA). *Agrobacterium* cultures containing each of the V_H_H-Fc, SC and JC constructs were resuspended in infiltration media, pooled in a 4:1:1 ratio, and co-infiltrated into leaf tissue. Crude extracts were immunoprecipitated with the anti-FLAG antibody specific to the SC subunit, then separated and detected on a western blot probing for either anti-cmyc (V_H_H-Fc subunit), or anti-HA (JC subunit). Bands matching the predicted 44 kDa size of V_H_H-Fc were detected with anti-c-myc antibody in crude extract transformed with V_H_H-Fc, V_H_H-(5 + 1) Fc, co-expressed V_H_H-Fc/SC/JC and co-expressed V_H_H-(5 + 1)Fc/SC/JC, but no bands were detected in crude extract expressing only JC or SC (Figure 3A). After co-IP, ∼44 kDa bands were seen only in extracts co-expressing V_H_H-Fc/SC/JC and V_H_H-(5 + 1)Fc/SC/JC (Figure 3B). This suggests that both SC and V_H_H-Fc or SC and V_H_H-(5 + 1)Fc interact, and that the mutations in Fc did not hinder this interaction. The size mismatch between the observed band of ∼50 kDa and the predicted size of ∼44 kDa may possibly either be due to a gel shift from the effect of SDS on the tertiary structure (Rath et al., 2009). A laddering effect showing several bands of higher molecular weight may suggest the presence of folding intermediates of the complex or alternatively may be a product of aggregation. Similarly, detection with anti-HA indicated bands of ∼20 kDa, matching the predicted size of JC, in crude extract transformed with JC, V_H_H-Fc/SC/JC and V_H_H-(5 + 1)Fc/SC/JC (Figure 3C). After Co-IP, ∼20 kDa bands were seen only for the co-expressed V_H_H-Fc/SC/JC and V_H_H-(5 + 1)Fc/SC/JC, indicating that SC and JC are present in the same complex (Figure 3D).

### Engineered V_H_H-(5 + 1)Fc Retains the Ability to Bind and Neutralize EHEC Strains O157:H7, O26:H11, O145:Hnm, and O111:Hnm

**FIGURE 4:**
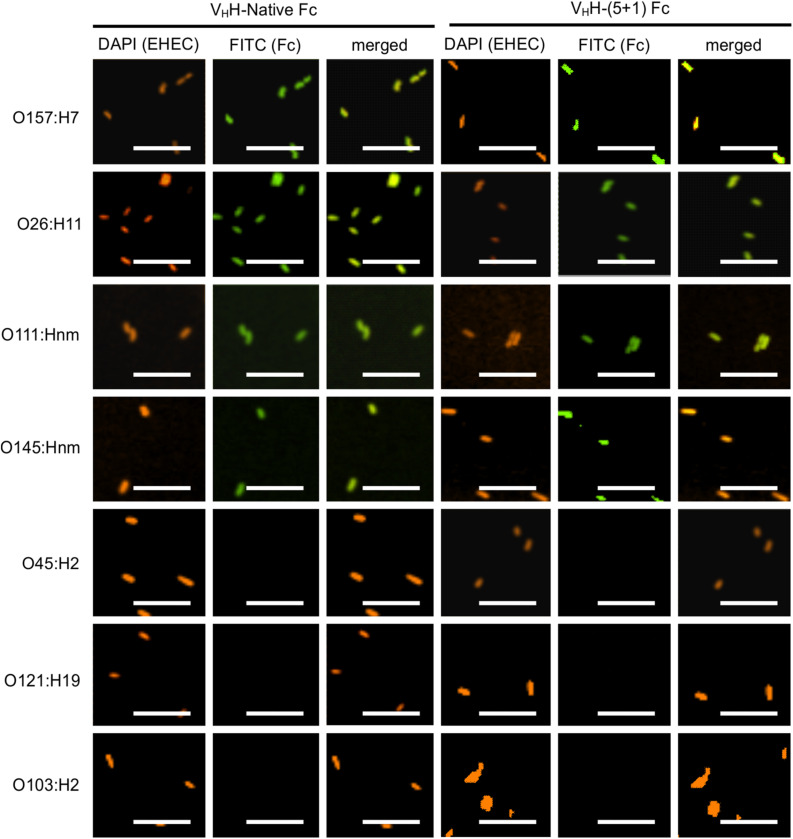
V_H_H-(5 + 1) Fc retains the same binding pattern across EHEC strains as V_H_H-Native Fc. Shown are confocal images of the seven most prevalent EHEC strains incubated with either V_H_H-Native Fc or V_H_H-(5 + 1) Fc. DAPI was used to visualize EHEC cells (orange) and a FITC-conjugated antibody (green) was used to immunolabel the Fc specifically. Size bar = 10 μm

To determine if the rationally designed mutations impact V_H_H-Fc’s pattern of cross-serotype binding to EHEC, we used a FITC conjugated secondary antibody to track the binding of V_H_H-Fc and V_H_H-(5 + 1)Fc to bacterial cell surfaces. We saw consistent co-localization of FITC signal with strains O26:H11, O145:Hnm, O111:Hnm and O157:H7 cells for both V_H_H-Fc and V_H_H-(5 + 1)Fc (Figure 4). Both V_H_H-Fc and V_H_H-(5 + 1)Fc do not seem to bind O45:H2, O121:H19 and O103:H2. We have previously shown by a sequence alignment of the extracellular residues of intimin that this pattern of binding may be explained by greater sequence similarity across strains O26:H11, O145:Hnm, O111:Hnm and O157:H7 and conversely, greater divergence for strains O45:H2, O121:H19 and O103:H2, presumably at antigenic sites to which the V_H_H binds (Saberianfar et al., 2019). As a negative control, EHEC cells were also treated with PBS instead of antibodies and similarly stained but did not show fluorescence under FITC-related imaging conditions (480 nm excitation and 520–540 nm detection).

**FIGURE 5:**
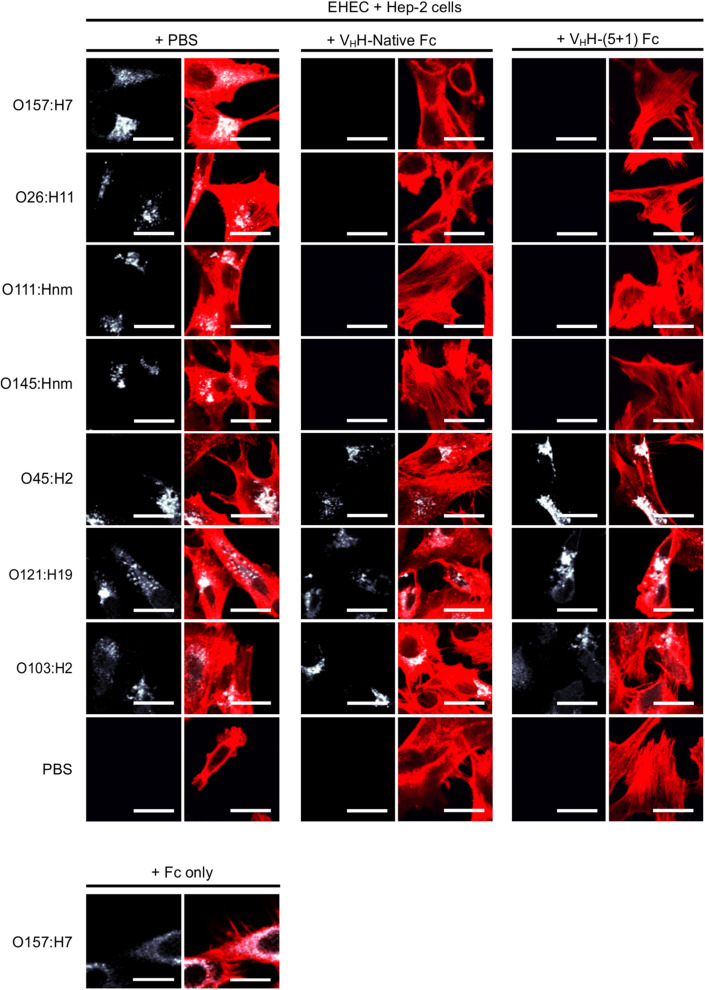
V_H_H-(5 + 1)Fc retains the V_H_H-Native Fc’s pattern of cross-serotype protection by neutralizing bacterial levels of O26:H11, O111:Hnm, O145:Hnm and O157:H7. Shown are confocal images of the seven most prevalent EHEC strains (white) that have been incubated with HEp-2 cells (red) in the presence of either 1x PBS as a control, with V_H_H-Native Fc or with V_H_H-(5 + 1)Fc. As a control against non-specific Fc binding, O157:H7 was incubated with Fc only to confirm that neutralization was mediated through the V_H_H. Size bar = 20 μm

**FIGURE 6:**
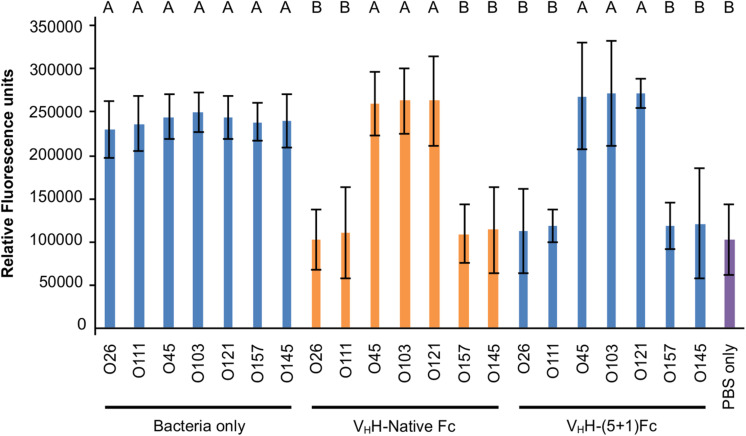
V_H_H-(5 + 1)Fc neutralizes bacterial levels of O26:H11, O111:Hnm, O145:Hnm and O157:H7 to baseline levels similarly to V_H_H-Native Fc. Shown is the relative fluorescence of seven EHEC strains that were immunolabelled, incubated on HEp-2 cells and treated with either V_H_H-Native Fc, V_H_H-(5 + 1)Fc or with 1x PBS as a positive control. As a negative control, HEp-2 cells were incubated with 1x PBS without addition of a bacterial strain or antibody. Letters indicate a significant difference of the amount of immunolabelled adherent bacteria as determined by a one-way ANOVA with a *post hoc* Tukey HSD test (*p* < 0.05, *N* = 3 biological replicates). Error bars indicate standard error of the mean.

Intimin, the antigenic target of the V_H_H-Fc, mediates the intimate attachment of EHEC to epithelial cells. We previously showed that V_H_H-sIgA neutralizes EHEC’s ability to intimately adhere to epithelial cells *in vitro* (Saberianfar et al., 2019). Here, we investigated if the V_H_H-Fc alone is sufficient to neutralize EHEC’s ability to adhere to human epithelial type 2 (HEp-2) cells, and if the rationally designed mutations impacted the V_H_H-Fc’s pattern of cross-serotype protection. HEp-2 cells have previously been shown to be an appropriate model system for intimin-based adherence and colonization (McKee et al., 1995). Compared to the respective positive controls of no V_H_H-Fc (+ PBS treatment), the addition of either V_H_H-Fc or V_H_H-(5 + 1)Fc seemed to abrogate the adhesion of EHEC strains O26:H11, O111:Hnm, O145:Hnm and O157:H7 to HEp-2 cells (Figure 5). Similarly to the binding assay, both V_H_H-Fc and V_H_H-(5 + 1)Fc showed no observable effect on inhibiting adhesion for strains O45:H2, O103:H2 and O121:H19. As a control to show that neutralization is specifically mediated by the V_H_H, the Fc without an attached V_H_H was incubated with O157:H7 and HEp-2 cells and showed a similar degree of adherent bacteria, based on microscopy and relative fluorescence, as did incubation without antibody (Figure 5). To quantify the relative neutralization capacity of the V_H_H-Fc compared to the V_H_H-(5 + 1)Fc, we adapted the adhesion assay for fluorometry and measured the relative fluorescence of HEp-2 cells incubated with a culture of each of the seven EHEC strains with and without either V_H_H-Fc or V_H_H-(5 + 1)Fc. The addition of either antibody showed the same pattern of reducing the relative fluorescence caused by adherent bacteria for strains O26:H11, O111:Hnm, O145:Hnm and O157:H7 to background levels (Figure 6). Similar to the binding assay, strains O45:H2, O121:H19 and O103:H2 showed no neutralization.

## Discussion

In this study, we engineered a bovine IgA Fc to improve its accumulation alone, and when fused to a V_H_H, by a combination of five supercharging residues and a disulfide pair to tether an unstructured domain at the C-terminal. Our initial motivation for engineering the Fc was to explore if it could be a practical solution for the problem of heterologous mAb yield in plant platforms, which has been a primary limiting factor for advancing many therapeutics toward market. Conceptually, engineering for protein supercharging relies on an inverse relationship that exists between protein charge extent and aggregation propensity (Carballo-Amador et al., 2019). The concept of ‘resurfacing’ a protein has recently been of interest in the context of biobetter development because substitutions to protein surfaces are usually better tolerated than buried side chains and folding is driven primarily by the loss of solvation of the core hydrophobic residues (Dill et al., 2008; Chapman and McNaughton, 2016). Indeed, because surface side chains do not become buried and retain a similar environment in both a folded versus unfolded state, solvent-exposed amino acids are thought to contribute less energy toward stabilizing the folded state compared to buried core residues (Tokuriki et al., 2007). Our results indicate that each of these supercharging substitutions, along with disulfide mutations, can individually improve *in planta* accumulation of either the Fc or V_H_H-Fc by as much as four- and six-fold respectively (Fc: 175.17 ± 20.22 mg/kg FW; V_H_H-Fc: 52.38 ± 1.40 mg/kg FW), and then be combined for an incremental increase [(5 + 1)Fc: 599.47 ± 25.40; V_H_H-(5 + 1)Fc: 236.19 ± 41.32]. Regarding market accessibility, these mutations permit overcoming of the rule of thumb benchmark of 100 mg/kg FW (Rybicki, 2009; Schillberg et al., 2019). We have previously found the yield of the native V_H_H-Fc construct to reach as high as 220 mg/kg FW when expressed using the pEAQ-DEST vector (Saberianfar et al., 2019) whereas in this study, we obtained only 12.5 mg/kg FW for the same construct when expressed using the pCaMGate vector. There are several differences in vector features that may possibly account for this difference in accumulation. In particular, the pEAQ vector contains modified 5′ and 3′-untranslated regions from cowpea mosaic virus (CPMV) RNA-2 that were shown to enhance the level of expression of the gene they flank (Sainsbury and Lomonossoff, 2008). As well, the pEAQ vector permits multi-gene expression of both the native V_H_H-Fc and p19, a suppressor of post-transcriptional gene silencing (Silhavy et al., 2002). A multi-gene vector may allow for a more thorough infiltration of both constructs into plant cells compared to co-infiltrating Agrobacteria containing different vectors, as done in the current study, and thus the lower accumulation may reflect a greater degree of post-transcriptional gene silencing (Westerhof et al., 2016). Although the absolute accumulation levels are lower with the pCaMGate vector, the relative yield increase from engineering the Fc is nonetheless meaningful as a yield improvement technique and we expect the mutations we have introduced in the bovine IgA Fc to lead to higher accumulation than reported by Saberianfar et al. (2019) when using the pEAQ vector. Previous studies using IgG Fc in bacterial systems have found similar success in promoting mAb stabilization by supercharging, particularly substitutions using aspartic and glutamic acid (Dudgeon et al., 2012; Buchanan et al., 2013; Lee et al., 2013; Courtois et al., 2015). This study is the first report demonstrating that this strategy can be successfully implemented for an IgA Fc in a plant platform.

Of the six supercharging candidates tested, five improved yield. The failure of the sixth to improve yield may be due to an incorrect prediction of surface exposure of that residue. If that residue was buried then its mutation to aspartic acid would not be expected to have an effect on surface charge and thus an effect on aggregation. Regarding the appropriateness of using a predicted structure for rational design, a previous validation study of I-TASSER on *de novo* protein design of 87 proteins showed that I-TASSER could accurately fold the designed sequences with an average root mean square deviation (RMSD) of 2.1Å (Mitra et al., 2013). Previous studies have also used I-TASSER for rationally designing stability into a variety of proteins (Wan et al., 2019; Guo et al., 2019; Ashfaq et al., 2021). Still, the resolution of the model is a key limitation on the success rate of the designed screen whereby inaccurately estimated surface exposure of asparagine and glutamine residues or the Cα distance of neighboring cysteines directly affects the prediction of supercharging or *de novo* disulfide mutations respectively. In another related project, we performed a similar screen on an EHEC subunit vaccine and found that using two supercharging candidates for an EHEC subunit vaccine improved yield by ∼20% when produced in the chloroplast (Chin-Fatt, unpublished). Notably, this protein was both intrinsically disordered and a membrane protein. Thus, this rational design approach may potentially be broadly applicable for other plant produced recombinant proteins.

We also designed a novel disulfide bridge in the CH3 domain of the Fc to fasten the C-terminal end of strand G to the N terminal end of strand F. In the disulfide screen, only two of the six disulfide candidates showed improved yield. Incorrect disulfide mutants are known to be strongly destabilizing because free thiol groups of unpaired cysteines are highly reactive when folding in the ER leading to intra-molecular disulfide scrambling, covalent oligomerization and consequently degradation (Trivedi et al., 2009). However, in a similar study, a *de novo* disulfide bond that tethered two β strands in two opposite β sheets of a variable heavy chain domain was proposed as a general strategy to increase thermal and conformational stability in nanobodies without compromising functionality (Saerens et al., 2008). Because nanobodies also tend to preserve the Ig-like fold, this may suggest a broader applicability of disulfide based domain tethering for stabilization of Greek key proteins. Interestingly, the study’s novel cysteine pairs were also not able to be predicted by algorithms, including Disulfide by Design, and were instead, like for G196C/R219C, identified based on manual inspection of the Cα distance in crystal structures and side chain orientation.

Another protein engineering approach for overcoming low yields of plant-made antibodies was based on exchanging sequences in the variable heavy chain domains of separate antibodies that showed differential accumulation (Zischewski et al., 2016). After transient expression in leaves of *N. benthamiana*, yield increased for several chimeras ranging from ∼250 mg/kg FW to as high as 2,000 mg/kg FW. Their analysis of the transcript and protein levels of the native and chimeric antibodies also suggested that the main events limiting yield were either translational or post-translational in nature. So, to address low yields of plant-made antibodies, the main research focus should arguably be to stabilize the protein folded state, such as by the rational design of supercharging and *de novo* disulfides. Another approach for stabilizing a murine IgG in *Nicotiana tabacum* has been to mutate protease-sensitive sites in the antibody light and heavy chains to increase proteolytic resistance (Hehle et al., 2016). Alteration of these sites was shown to enrich for the full-size antibody and to reduce the accumulation of fragments from proteolytic degradation. The negative impact of endogenous proteases on the yields of plant recombinant proteins targeted to the secretory pathway in *N. benthamiana* is well documented (Benchabane et al., 2010; Robert et al., 2013; Jutras et al., 2020). Although targeting sequence specific cleavage sites is important, our results suggest that degradation by proteolysis at unstructured regions may not only be substantial but also may be mitigated by appropriate disulfide tethering.

Co-immunoprecipitation of the differentially tagged subunits that had been co-expressed indicated that engineering of the Fc did not impact its capacity to assemble with the JC and SC subunits to form the secretory complex. Although the JC can double the avidity, and thus the potency, and the SC can protect the complex from proteolytic attack, full assembly comes at the cost of yield (Saberianfar et al., 2019). We had previously characterized the binding and neutralization of a separate anti-EHEC V_H_H-sIgA because it was previously thought that the entire complex was needed for protection as the secretory form is the predominant functional unit in mammals (Saberianfar et al., 2019). Although these V_H_H-sIgA’s are effective, accumulation is apparent as multiple assembly intermediates and, if selecting for only the final multi-subunit assembly, yield is diminished. Other groups have observed such a reduction for murine, chicken and human sIgA in plants with a significant proportion of monomeric IgA and assembly intermediates occurring more so than fully assembled sIgA (Ma et al., 1995; Wieland et al., 2006; Juarez et al., 2013; Paul et al., 2014). A possible reason for this is a cryptic sequence in the tailpiece targeting the Fc to the vacuole before disulfide formation with the JC can be accomplished (Frigerio et al., 2000; Hadlington et al., 2003). Indeed, joining chain incorporation has been shown to be a major limiting step for assembly (Westerhof et al., 2016). Also, the presence of assembly intermediates may be due to sporadic transformation of plant cells such that expression of the fully assembled sIgA may be limited to occurring only in the cells receiving all transgenes. Indeed, expression of the fully assembled sIgA has been shown to be enriched using a multi-gene vector (Westerhof et al., 2016). Recently, an anti-F4 V_H_H-Fc monomer was shown to be effective in protecting piglets against F4 ETEC challenge suggesting that the assembled secretory format may not be necessary for protection (Virdi et al., 2019). The use of a single transgene, rather than three, simplifies production, improves yield and is still protective in animal models. So, with yield as a priority, we opted to focus on the simplified monomer to characterize V_H_H functionality.

We also showed that engineering of the Fc did not impair the V_H_H’s pattern of cross serotype binding and neutralization for strains O26:H11, O111:Hnm, O145:Hnm and O157:H7. Collectively, these strains account for ∼72% of all EHEC-related illnesses, affecting an estimated 1,158 per 100,000 individuals in the U.S. (CDC, 2017). Testing for cross-serotype protection is important because the vast majority of EHEC surveillance and therapeutic development has historically focused on O157:H7 only, despite the growing importance of non-O157 strains. Based on surveillance of agricultural sites across Canada, the prevalence of non-O157:H7 strains now exceeds that of O157:H7 by about 7% (Public Health Agency of Canada, 2015). Currently, there are no EHEC diagnostics or antibody therapeutics in the market or in the pipeline that have been shown to detect O157:H7 as well as non-O157:H7 strains.

In this study we demonstrated that rationally designing an IgA Fc for supercharged residues and *de novo* disulfides can impart a significant improvement of *in planta* accumulation without sacrificing the binding efficacy of the V_H_H or the Fc’s ability to structurally assemble with other secretory subunits. Given that combining these mutations did not seem to destabilize the protein, it is tempting to speculate on identifying both the upper limit of mutation that can be accommodated as well as an exhaustive list of all relevant mutations in order to fully optimize accumulation. Based on a stability estimating algorithm across globular proteins, about 70% of amino acid mutations are neutral and 20% are significantly destabilizing (Tokuriki et al., 2007). Within the scope of all possible amino acid variations of the Fc being 1.15 × 10^48^, isolating stabilizing mutants by systematic or random mutation is not practical. Therefore, relevant techniques to identify stabilizing mutations are required. With regards to combining mutations, supercharging has been used to alter the net charge of GFP by as much as 55 charge units without compromising protein folding or function and enabling significant resistance to aggregation (Lawrence et al., 2007). Also, various separate rational design strategies have been combined for improving protein stability without compromising function (Courtois et al., 2016; Fei et al., 2013). Given that we were able to combine supercharging and disulfide mutations without noticeably compromising function, this raises questions of whether this strategy can be combined with other strategies to further improve yield or if there is a theoretical mutational load that can be tolerated within the context of the plant cell as a heterologous environment. Because it folds independently from the V_H_H, this engineered IgA Fc scaffold may potentially be useful as a modular tool for improving accumulation of other V_H_Hs as well. Future experiments will seek to test how universally applicable (5 + 1)Fc is as a scaffold. Although plant-based IgA production is still an emerging field, we are optimistic that such a tool could be of value in overcoming the yield hurdles that have thus far hindered transition of plant-based antibody therapeutics to market.

## Materials and Methods

### Design and Selection of Rationally Designed Fc Candidates

Estimating of negatively supercharged Fc candidates was performed computationally by first ranking residues for solvent accessibility by their average number of neighboring atoms (within 10 Å) per side-chain atom (AvNAPSA) and then identifying highly polar solvent-exposed Asn and Gln residues for mutation to their negatively charged counterparts, Asp and Glu respectively (Schrodinger, 2010). For visualizing the multi-subunit complex, the V_H_H-Fc sequence was submitted as a dimer to the SPRING server which uses a template-based threading algorithm across the PDB library to predict structure and assembly (Guerler et al., 2013)

Disulfide candidates were selected by manual inspection of the model in PyMol (Schrodinger, 2010) based on residue proximity (under 5 Å) or by ranked selection of ∑B-factor, a measure of dynamic mobility for each atom, in the DisulfidebyDesign 2.0 software (Craig and Dombkowski, 2013). To retain the functionality of the native Fc, native disulfide sites were avoided (inter-chain: 16-18, 205-207, 199-201, 13-15; intra-chain: 100-102, 271-273, 412-414, 601-603; tailpiece to JC: 718-720; 235-237; free S-H: 235-237).

### Cloning of Rationally Designed Mutations and Transient Expression in Plants

The bovine Fc, JC and SC sequences were obtained from the NCBI public database (ANN46383, NP_786967 and NP_776568 respectively) and synthesized by Bio Basic Inc. (Markham, ON, Canada). Rational design mutations were individually made to the native bovine IgA Fc sequence using an *in vitro* single primer site-directed mutagenesis method (Huang and Zhang, 2017). Combination of mutations was done using a multi-site-directed mutagenesis method (Liang et al., 2012). Genetic fusion to an anti-EHEC V_H_H (denoted as V_H_H9 identified previously in Saberianfar et al., 2019) was done using a sequence and ligation independent cloning (SLIC) method (Li and Elledge, 2007). All cloning was confirmed by sequencing.

To enable expression in leaf tissue, each Fc and V_H_H-Fc construct was cloned into an in-house pCaMGate plant expression vector (Pereira et al., 2014) using the Gateway^®^ cloning kit (Thermo-Fisher Scientific Inc., Waltham, MA, United States). The pCaMGate vector attaches a PR1b tobacco signal peptide for targeting the protein to the secretory pathway, an N-terminal Xpress tag for protein stability, a C-terminal c-myc tag for detection and purification as well as a C-terminal KDEL tag for retrieval to the ER of the plant cell. The JC and SC sequences were cloned into pEAQ-DEST-1 expression vectors (Sainsbury et al., 2009) along with C-terminal HA and FLAG tags respectively (Saberianfar et al., 2019). Vectors were then transformed into *Agrobacterium tumefaciens* (EHA105) and transformed bacteria were selected, using kanamycin and rifampicin antibiotics. Cultures were resuspended into infiltration media consisting of Gamborg’s B-5 medium solution (3.2 g/L Gamborg’s B5 salts with vitamins, 20 g/L sucrose, 10 mM MES, pH 5.6, 200 μM acetosyringone). Each construct was co-infiltrated with a vector carrying p19, a suppressor of post-transcriptional gene silencing from *Cymbidium* ringspot virus (Silhavy et al., 2002), each at a final optical density (OD_600_) of 0.3. Transient expression was performed by syringe infiltration into leaf tissue of *N. benthamiana* plants. Plants were grown in a growth chamber at 22°C with a 16 h photoperiod at a light intensity of 110 μmol m^–2^ s^–1^ for 7 weeks and fertilized with water soluble N:P:K (20:8:20) at 0.25 g/L (Plant products, Brampton, ON, Canada).

### Protein Extraction and Western Blotting

Pre-weighed leaf samples were frozen in liquid nitrogen and homogenized with silica beads (Bio Spec Products Inc., Bartlesville, OK, United States) for 2 min using a TissueLyser II (Retsch Inc., Newton, PA, United States). One mL of either a denaturing extraction buffer (1x PBS, pH 7.5, 4% SDS, 2% PVPP) or a native buffer (1x PBS, pH 7.5, 0.1% Tween-20, 1 mM EDTA, 2% PVPP, 100 mM sodium ascorbate, 8 M sucrose, 1 μg/mL leupeptin, 1 mM PMSF, 1 μg/mL pepstatin A) was added per approximately one hundred mg of sample. All samples were vortexed on high speed for 30 s and centrifuged at 20,000 x *g* for 10 min to remove insoluble debris. Extracted proteins were combined with 1/5^*th*^ volume of 5 x reducing loading buffer (0.3 M Tris-HCl pH 8.0, 5% SDS, 10% glycerol, 100 mM DTT, 0.05% Phenol Red) heated at 90°C for 10 min, then loaded onto Express Plus 4-20% gradient polyacrylamide gels (Genscript Inc., Piscataway, NJ, United States). Gels were run at 100 V for 100 min, then transferred to polyvinylidine difluoride (PVDF) membrane using the Trans-Blot Turbo transfer system (Bio-Rad Laboratories Inc., Hercules, CA, United States). Blots were blocked overnight with 5% skimmed milk in tris-buffered saline, pH 7.5, and proteins of interest were probed with a mouse anti-c-myc antibody (diluted 1:1,000; Genscript Inc., Piscataway, NJ, United States) and the One-Hour Basic western kit for mouse primary antibody (Genscript Inc., Piscataway, NJ, United States). Detection was performed using Amersham ECL western blot detection reagents (GE Healthcare, Mississauga, ON, Canada) or Enhanced Chemiluminescent detection solution (Biorad Laboratories Inc., Hercules, CA, United States) and a MicroChemi 4.2 imaging system with GelCapture acquisition software (DNA Bio-Imaging Systems Ltd., Jerusalem, Israel). For staining, membranes were rinsed in methanol followed by ultrapure water, stained using GelCode Blue (Thermo-Fisher Scientific Inc., Waltham, MA, United States) for 15 min, and destained in 50% methanol 1% acetic acid for 15 min. Quantification of accumulation was done by densitometry using a calibrated standard curve of an in-house-produced purified protein. Statistical significance for accumulation in tissue expressing the native and mutant Fc was determined using a one-way ANOVA with three to five biological replicates. *Post hoc* comparisons were then performed on the accumulation means using the Tukey HSD test.

### Recombinant Protein Purification

Plant extracts were prepared under native conditions as described above. All samples were vortexed on high speed for 30 s and centrifuged twice at 20,000 × *g* for 10 min to remove insoluble debris. Purification on the supernatant was performed by affinity chromatography using an anti-c-myc mild purification kit (MBL International Corp., Woburn, MA, United States) according to the manufacturers’ protocol. Briefly, 100 μL anti-c-Myc tag bead suspension was added to 3 mL of clarified extract and incubated at 4°C for one hour using an end-over-end shaker. The extract was then added to a spin column and centrifuged for 10 s. The beads were then washed three times and the protein eluted using a c-Myc tag peptide in 1x PBS.

### Enterohemorrhagic *E. coli* Binding and Neutralization Assays

Enterohemorrhagic *E. coli* strains O26:H11, O45:H2, O103:H2, O121:H19, O111:Hnm and O157:H7 were obtained from Dr. Michael Mulvey at the Public Health Agency of Canada, National Microbiology Laboratory, *E. coli* Unit, Enteric Diseases Program, Winnipeg, MB. EHEC strain O145 (C625) was obtained from the American Type Culture Collection supplied by Cedarlane Labs in Burlington, ON. Strains were stored at −80°C in a level 2 containment laboratory. All experiments with EHEC were conducted in a level 2 containment laboratory. For propagation of live cultures, single colonies were picked and inoculated in Luria-Bertani (LB) medium overnight and then subculture into pre-warmed Dulbecco’s Modified Eagle Medium (DMEM) (Thermo-Fisher Scientific, Cat. No. 10566016) at a 1: 50 dilution and incubated at 37°C in 5% CO_2_ for 2 h without shaking. Binding and neutralization assays were performed as previously described (Saberianfar et al., 2019).

To assess binding, we incubated either V_H_H-Fc or V_H_H-(5 + 1)Fc with the seven EHEC strains recognized as food adulterants, EHEC O26:H11, O45:H2, O103:H2, O145:Hnm, O121:H19, O111:Hnm or O157:H7. After washing with PBS and fixing with 2.5% paraformaldehyde, we visualized bacteria with DAPI and V_H_H-Fc binding using a secondary fluorescent antibody (rabbit anti-bovine-FITC) (1:40 dilution, Thermo-Fisher Scientific, Cat. No. SA1-36043) that binds Fc. The cells were then dried onto poly-L-lysine coated coverslips (Millipore Sigma, Cat, No. S1815) and mounted onto glass slides with Aqua-Poly/Mount (Polyscience Inc., Warrington, PA, United States, Cat. No. 18606). To visualize co-localization, FITC and DAPI sequential imaging was performed with an Olympus LSM FV 1200 using a 100x oil objective lens. FITC fluorescence was excited using a 480 nm laser and detected at 520–540 nm. DAPI fluorescence was excited at 350 nm and detected at 455–465 nm.

To assess neutralization, eight-well chamber slides were seeded with ∼2 × 10^5^ human epithelial-2 (HEp-2) cells (American Type Culture Collection) in DMEM supplemented with 10% fetal bovine serum (FBS) and incubated overnight at 37°C in 5% CO_2_. For the assay, the DMEM was removed from the culture and replaced with 225 μL fresh DMEM. HEp2 cells were then incubated with 25 μL of an overnight starter culture of one of seven EHEC strains (O26:H11, O45:H2, O103:H2, O145:Hnm, O121:H19, O111:Hnm and O157:H7) in the presence or absence of either the V_H_H-Fc or V_H_H-(5 + 1)Fc. Cells were then washed in 1x PBS to remove any non-adherent bacteria, fixed in 2.5% paraformaldehyde and then visualized using fluorescent actin staining with Alexa 647 rhodamine phalloidin (red) (Thermo-Fisher Scientific, Cat. No. A22287) on HEp-2 cells and a donkey anti-rabbit alexa 350 secondary antibody (blue) (Thermo-Fisher Scientific, Cat. No. A10039) that specifically hybridizes to EHEC cells. Cells were then washed in 1x PBS and mounted using AquaPoly/Mount (PolyScience Inc., Warrington, PA, United States, Cat. No. 18606). To visualize adherence to HEp-2 cells, sequential imaging was done using an Olympus LSM FV 1200 and a 64x water objective lens. Alexa 647 phalloidin was excited at 650 nm and detected at 660 nm-680 nm. The donkey anti-rabbit Alexa 350 antibody was excited at 350 nm and detected at 455-465 nm. To quantify HEp-2 adherence inhibition by fluorometry, the assay was adapted by growing the HEp-2 cells in 96-well black fluorometry plates that had been previously coated with polyD-lysine, which enables cell adherence. Incubation, washing and immunolabelling was done similarly as above. Relative fluorescence was then measured using a Synergy2 plate reader (Biotek, Winooski, United States) using the Gen5 v1.10 software (Biotek). Wells were measured at 37°C with 5 s intermediate shaking with the same excitation and detection conditions as above.

## Data Availability Statement

The original contributions presented in the study are included in the article/Supplementary Material, further inquiries can be directed to the corresponding author/s.

## Author Contributions

AC-F and RM conceived the study. AC-F performed the experiments and wrote the manuscript. AC-F, RS, and RM edited the manuscript. All authors contributed to the article and approved the submitted version.

## Conflict of Interest

The authors declare that the research was conducted in the absence of any commercial or financial relationships that could be construed as a potential conflict of interest.
