# Supplementary material for: A Rationally Designed Bovine IgA Fc Scaffold Enhances in planta Accumulation of a VHH-Fc Fusion Without Compromising Binding to Enterohemorrhagic E. coli
Source: Front Plant Sci. 2021 Apr 14;12:651262. doi: 10.3389/fpls.2021.651262 (PMC8079772; doi:10.3389/fpls.2021.651262)
Supplement: Supplementary file 1 [file Image_1.PDF]

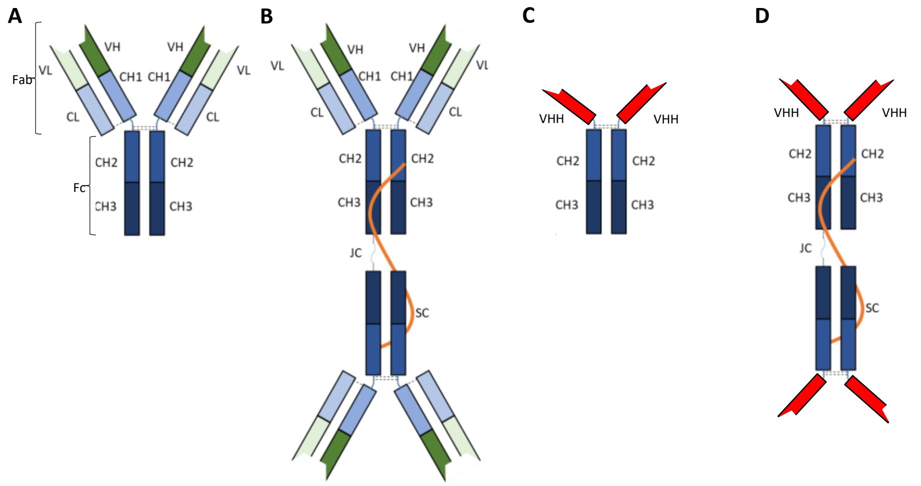

Supplementary Figure 1. Schematic showing the generalized structure of different IgA formats. VH: Variable Heavy chain, VL: Variable Light chain, CH: Constant Heavy chain, CL: Constant Light chain, JC: Joining Chain, SC: Secretory Component, Fab: antigen binding fragment, Fc: Fragment crystallizable, V_HH: single domain antibody consisting of the variable heavy chain only. Dotted lines represent interchain disulfide bonds

A) The native immunoglobulin A (IgA) is a tetrameric molecule comprising two heavy chains and two light chains. Each heavy chain consists of a variable domain (VH) and three constant domains (CH1, CH2 and CH3) whereas each light chain consists of one variable domain (VL) and one constant domain (CL). Cleavage of the hinge separating CH1 and CH2 yields two separate portions: a Fab region and a Fc region. The Fab region, containing two variable and two constant domains, is generally considered as the antigen binding portion of the IgA. The Fc region, containing the dimerized constant domains CH2 and CH3, enables various effector functions and is required for assembly into the secretory complex.

B) The native secretory IgA complex (sIgA) consists of two copies of an IgA that are assembled with two additional strands, a JC and a SC. The JC connects the two IgA copies end-to-end and forms an interchain disulfide bond with a tailpiece extending from the CH3 domains of each IgA. The SC wraps around this paired structure and forms an interchain disulfide bond with a CH2 domain of each IgA.

C) The chimeric fusion of a V_HH with the Fc (V_HH-Fc) involves replacing the Fab region with a V_HH sequence. The result is a dimer of two heavy chains whereby each heavy chain consists of a variable region (V_HH) and two constant domains (CH2 and CH3).

D) The V_HH-Fc can also assemble into a secretory complex (V_HH-sIgA). Like the native secretory complex, V_HH-sIgA assembles as two copies of the V_HH-Fc along with the JC and SC. Assembly with the JC and SC requires the CH3 and CH2 domains respectively on the Fc.
