# Supplementary material for: A Rationally Designed Bovine IgA Fc Scaffold Enhances in planta Accumulation of a VHH-Fc Fusion Without Compromising Binding to Enterohemorrhagic E. coli
Source: Front Plant Sci. 2021 Apr 14;12:651262. doi: 10.3389/fpls.2021.651262 (PMC8079772; doi:10.3389/fpls.2021.651262)
Supplement: Supplementary file 2 [file Data_Sheet_1.pdf]

Supplementary Table 1. Rational design mutations that were tested for improved yield and their underlying design strategies. Each mutational candidate was transiently expressed in leaves of *N. benthamiana* and yield assessed based on western blot densitometry of harvested leaf tissue.

**Mutation**	**Strategy**	**Improved yield?**
T113C/A207C	*de novo disulfides*	n
K146C/T179C	*de novo disulfides*	n
G196C/R219C	*de novo disulfides*	y
E132C/D189C	*de novo disulfides*	n
G86C/K108C	*de novo disulfides*	n
Pre-existing C10/K40C	*de novo disulfides*	y
N9D	supercharging	y
N84D	supercharging	y
N131D	supercharging	y
N156D	supercharging	n
Q175E	supercharging	y
Q195E	supercharging	y

Disulfide candidates were chosen either from manual selection based on neighbouring Cα distance and side chain orientation as visualized in PyMol in order to tether two particular strands, or based on a thermodynamic estimate from the Disulfide by Design 2.0 software. Specifically for manual inspection, K146C/T179C was chosen to tether the N terminal of strand E to the N terminal of strand C in the CH3 domain, G196C/ R219C was chosen to tether the N terminal of strand F to the C terminal of strand G in the CH3 domain, E132C/D189C was chosen to tether the C terminal of strand A to the C terminal of strand C in the CH3 domain and G86C/K108C was chosen to tether the N terminal of strand F to the C terminal of strand G in the CH2 domain. A similar strategy of domain tethering has been suggested for human IgG Fc (Ruker et al., 2012). Specifically for choices for Disulfide by Design 2.0, K40C/pre-existing cysteine at position 10 was chosen based on a high positive sum of B factors and T113C/A207C was chosen based on a high positive change in energy (kcal/mol).

Supplementary Table 2. Predictions of stabilizing disulfide mutations using Disulfide by Design 2.0.

Residue 1 (position)	Residue 2 (position)	Energy (kcal/mol)	ΣB-factor
CYS (6)	GLY (68)	3.83	11.41
**CYS (10)**	**LYS (40)**	**3.72**	**10.72**
PRO (12)	LEU (39)	3.39	7.56
PRO (19)	ALA (30)	1.18	5.74
SER (28)	ALA (80)	2.84	6.35
ALA (42)	GLU (43)	3.04	8.29
ASN (50)	SER (90)	3.31	5.63
PRO (51)	ALA (57)	6.36	6.85
THR (52)	GLN (87)	2.96	7.93
GLY (53)	PRO (82)	4.58	7.07
VAL (58)	GLN (59)	2.07	6.8
GLN (59)	GLY (60)	1.29	6.69
SER (61)	SER (74)	4.36	6.16
SER (92)	THR (103)	2.4	5.86
**THR (113)**	**ALA (207)**	**7.02**	**6.19**
PHE (114)	SER (144)	1.95	6
PRO (124)	GLU (127)	4.7	6.6
LEU (136)	LEU (186)	5.4	5.12
GLY (155)	THR (198)	5.62	6.04
LYS (163)	ARG (187)	3.55	6.19
SER (200)	THR (216)	3.09	5.28

The energy value (kcal/mol) is the enthalpic requirement for disulfide bond formation at that position. ΣB-factor is a thermodynamic estimate of the dynamic mobility of each atom. A greater positive ΣB-factor is associated with a greater increase of stability. Consideration of both parameters, ΣB-factor and energy value, has been recommended for the selection of novel disulfides (Dombkowski et al., 2013). Although C6/G68 is a higher ranked prediction using ΣB-factor, it was disregarded because C6 is involved in native disulfide formation. The selected mutations that were tested are shown in bold.

Supplementary Table 3. Accumulation of recombinant protein of mutational candidates in harvested leaf tissue. Candidates were transiently expressed in leaves of *N. benthamiana*, detected by western blotting and quantified by densitometry. n=3-5 biological replicates.

**Expression construct**	**Antibody accumulation (mg/kg FW)**	**SEM**	**Antibody accumulation (mg/kg FW)**	**SEM**	**Antibody accumulation (mg/kg FW)**	**SEM**
	4 dpi		6 dpi		8 dpi	
Fc (Native)	52.35	14.64	29.46	6.71	42.30	10.83
Fc (N9D)	159.96	18.57	102.55	30.44	114.55	8.95
Fc (N84D)	140.18	14.46	82.89	12.38	112.39	17.11
Fc (N131D)	183.89	11.72	111.79	22.33	136.10	11.65
Fc (Q175E)	176.23	14.95	112.40	20.12	136.38	13.08
Fc (Q195E)	226.22	26.03	141.30	21.78	175.17	20.11
Fc (Native)	14.82	6.51	16.51	6.67	12.18	6.32
Fc (K40C)	57.15	28.59	40.05	8.95	35.91	7.99
Fc (G196C/R219C)	30.22	4.20	138.43	23.89	132.02	26.63
Fc (Native)	36.58	5.10	29.91	3.98	23.91	4.08
Fc (N9D)	103.02	11.28	90.31	6.70	76.71	8.26
Fc (N9D/N84D/N131D)	244.04	12.36	233.07	15.59	213.33	13.22
Fc (N9D/N84D/N131D/ Q175E/Q195E)	320.44	20.78	313.16	16.74	296.18	18.48
Fc (G196C/R219C)	221.87	9.80	223.02	11.45	240.89	4.33
Fc (N9D/N84D/N131D/ Q175E/Q195E/G196C/R219C)	658.52	26.21	621.33	17.78	599.47	25.40
VHH-Fc (Native)	10.47	1.55	10.36	0.42	7.57	0.88
VHH-Fc (N9D)	33.00	4.72	37.02	4.13	26.42	4.96
VHH-Fc (N84D)	37.90	3.43	39.76	3.46	27.87	1.23
VHH-Fc (N131D)	34.58	2.42	35.27	1.51	27.82	1.97
VHH-Fc (Q175E)	36.47	1.83	40.47	2.90	34.53	2.90
VHH-Fc (Q195E)	43.73	2.61	49.13	3.47	40.71	1.29
VHH-Fc (G196C/R219C)	48.55	3.23	53.76	2.55	52.38	1.40
VHH-Fc (Native)					12.45	5.86
VHH-Fc (N9D)					28.67	11.32
VHH-Fc (N9D/N84D/N131D)					53.90	13.19
VHH-Fc (N9D/N84D/N131D/ Q175E/Q195E)					99.71	10.82
VHH-Fc (N9D/N84D/N131D/ Q175E/Q195E/G196C/R219C)					236.19	41.32

SEM= Standard error of the mean; FW=Fresh leaf weight
